# Semantic anomaly detection in school-aged children during natural sentence reading – A study of fixation-related brain potentials

**DOI:** 10.1371/journal.pone.0209741

**Published:** 2018-12-27

**Authors:** Otto Loberg, Jarkko Hautala, Jarmo A. Hämäläinen, Paavo H. T. Leppänen

**Affiliations:** 1 Department of Psychology, University of Jyväskylä, Jyväskylä, Finland; 2 Niilo Mäki Institute, Jyväskylä, Finland; Baycrest, CANADA

## Abstract

In this study, we investigated the effects of context-related semantic anomalies on the fixation-related brain potentials of 12–13-year-old Finnish children in grade 6 during sentence reading. The detection of such anomalies is typically reflected in the N400 event-related potential. We also examined whether the representation invoked by the sentence context extends to the orthographic representation level by replacing the final words of the sentence with an anomalous word neighbour of a plausible word. The eye-movement results show that the anomalous word neighbours of plausible words cause similar first-fixation and gaze duration reactions, as do other anomalous words. Similarly, we observed frontal negativity in the fixation-related potential of the unrelated anomalous words and in the anomalous word neighbours. This frontal negativity was larger in both anomalous conditions than in the response elicited by the plausible condition. We thus show that the brain successfully uses context to separate anomalous words from plausible words on a single letter level during free reading. From the P600 response of the scalp waveform, we observed that the P600 was delayed in the anomalous word neighbour condition. We performed group-level decomposition on the data with ICA (independent component analysis) and analysed the time course and source structure of the decomposed data. This analysis of decomposed brain signals not only confirmed the delay of the P600 response but also revealed that the frontal negativity concealed s more typical and separate N400 response, which was similarly delayed in the anomalous word neighbour condition, as was the P600 response. Source analysis of these independent components implicated the right frontal eye field as the cortical source for the frontal negativity and the middle temporal and parietal regions as cortical sources for the components resembling the N400 and P600 responses. We interpret the delays present in N400 and P600 responses to anomalous word neighbours to reflect competition with the representation of the plausible word just one letter different.

## Introduction

Forming a mental representation of the semantic content of text is the end goal of the reading process. This is achieved through a sequence of visual inputs and the analysis of these inputs. Exact features of this visual input sequence are modulated by the reading process itself. Indeed, previous research has shown that gaze behaviour, brain activation and the properties of processed text interact in a complicated manner [1,2]. Some attempts have been made to synthesise the knowledge obtained from separate eye-tracking and brain ERP (event-related potential) experiments, but fundamental differences in how these experiments are run impede the joint interpretation of the results. Studying eye-movement-locked brain activity (i.e. fixation-related brain responses) related to semantic processing during natural reading provides advances for the interpretation of both eye movements and brain responses. Specific methodological challenges arise from the dynamic nature of how eye movements occur during reading as well as the artefactual contamination of EEG from eye movements. However, the benefits of co-registering EEG and optical eye tracking in terms of increased ecological validity far outweigh the challenges, both of which are discussed below.

### Semantic processing of sentences in gaze behaviour and in brain activity

The semantic processing of sentences involves not only the processing of the individual words; the relationships between words also need to be considered to grasp the meaning contained in the sentences. It has been proposed that forming a representation of a sentence proceeds through three stages: 1) building a local phrase structure, 2) assigning syntactic and thematic and semantic relations and 3) integration to semantic representation [3]. Of particular interest here are stages 2 and 3.

Several factors affect eye movements during semantic processing. Predictability, for example, is a major determinant of fixation duration during reading [4]. In addition, the thematic relationships between words modulate the gaze behaviour. For example, when a word clearly violates the sentence context, it will result in a longer first-pass gaze duration (sum of fixation durations during the first pass of the target word) [5,6]. In earlier research merely implausible or improbable word in relation to thematic rules was not reflected in the first-pass measures but rather in the total fixation duration (sum of all fixation durations during the trial allocated to the target word), which indicates that implausibility is processed at a later stage than outright theme violation in the form of a semantic anomaly [5]. However more recent research with larger sample sizes and more suitable statistical approaches has shown that plausibility is capable of modulating first pass measures[7,8] and this includes influences from parafoveal processing[9,10].

A systematic way of inducing ERP effects reflecting semantic processing is to set up a semantic context with a sentence and then disrupt it by inserting a word that is anomalous with the established context. Consequently, this anomalous word violates the set of thematic rules established by the other words and gives rise to N400, a centroparietal brain response with negative polarity typically reaching its maximum amplitude in RSVP experiments at about 400ms post onset of the anomalous word [11]. N400 is thought to result from difficulty in integrating the meaning of the violating word with the context of the preceding sentence [11] or to reflect indexing of the goodness of fit of the currently processed word with the context of the sentence [12]. Both of these suggested functions are similar to stage 2 of the model described above [3]. Other factors besides congruency have also been shown to influence N400. For example word frequency affects the amplitude of N400 in low-context situations where prior context has not been defined, for example, at the beginning of the sentence. Thus, in order to avoid confounds from word frequency and to maximise semantic incongruity, the manipulation should be placed at the end of the sentence. Further, the presentation parameters of RSVP have direct consequences to N400. Faster presentation rates diminish amplitudes and shorten the latency of N400 [13], which emphasises the uncertainty regarding the ecological validity of RSVP experiments with slow presentation rates.

Generally, N400 experiments have manipulated only the semantic aspects without paying attention to low-level features, such as word length or visuo-spatial similarities of anomalous and plausible words. A fairly recent experiment [14] tested whether the deviation of a single letter from a probable word was sufficient to cause N400 –i.e. does the anomalous orthographic word neighbour of the predicted word cause a similar N400 as an anomalous word without a similar word body? N400 was found to be diminished for anomalous word neighbours compared to anomalous words without orthographic resemblance to the correct word, which was interpreted to indicate facilitated integration of words with similar word bodies. This effect was also present for pseudoword neighbours containing no semantic information, thus providing strong evidence that a set of orthographic features was pre-activated by the preceding context. As these findings were found in a fairly slow-paced (250ms stimulus duration + 250ms blank screen) RSVP design, the question remains as to whether a one-letter deviation from a plausible word would elicit similar effects during free reading.

Recently P600, a parietal positive ERP reaching maximal amplitude at 600ms that has been classically defined as a response to syntax violations [15], has also been shown to be elicited by semantic anomalies [3,16,17]. Similarly to N400, the P600 effects seem to diminish with faster presentation rates [18]. Curiously, P600 has been found to be related to regressive eye movements in an FRP study investigating the effects of free reading on brain activity during reading comprehension [1]. It was also found to be absent in the RSVP version of the experiment. This led the authors to conclude that coupling between P600 and regressions indicates attempts to recover or re-organise confusing content. It has also been argued that rather than encountering anomalies in a specific aspect of language, such as semantics, syntax or grammar, P600 reflects an integration phase or combinatory reprocessing of the elements of the sentence [3,19–21] or even a conscious-level perception of the whole sentence-level semantic anomaly [12].

The neural generators of N400 seem to be rather widespread [22,23]. Studies of individuals with brain lesions, studies with intracranial recordings and studies with magnetoencephalographic data have implicated contributions of the left and right temporal lobe, with a left hemispheric predominance [24]. The larger left than right hemispheric response can also be observed in a study comparing typically reading adults with dyslexic adults [25]. A recent study using a beamformer analysis of the magnetic equivalents of N400 (N400m) and P600 (P600m) found left superior temporal and posterior frontal regions to underlie N400m and distributed activation of bilateral frontal, posterior temporal and parietal regions P600m [26]. Further, recordings with functional magnetic resonance imaging (fMRI) suggest that the processing of semantic anomalies occurs in mid portions of the superior temporal region and insular cortex, both bilaterally, whereas syntactic processing is associated with the anterior portion of the left superior temporal sulcus and left posterior frontal operculum [27]. In positron emission tomography (PET) studies, the role of the angular gyrus has also been implicated in semantic processing [28,29]. It needs to be noted here that different measuring and analysis methods as well as different experimental designs have different sensitivities and can thus lead to quite distinct location results. Different results do not render conclusions right or wrong but rather reflect different aspects of the process, which are reflected in different signals.

In our current study, we investigated semantic processing and how orthographic similarity between anomalous and plausible words affects semantic processing during free reading in 12–13-year-old children using FRPs (co-registered eye tracking and EEG time-locked to fixation onset). We employed a free sentence reading paradigm to examine the effects of a semantic anomaly in two conditions: unrelated anomalous words and anomalous orthographic word neighbours of plausible words as the target words appearing at the end of the sentence. We were especially interested in whether a deviation of a single letter from a plausible word would elicit complementary effects on gaze behaviour in relation to brain activation during free reading. Based on previous studies [14], the anomalous orthographic word neighbours were expected to produce reduced N400 compared to the unrelated anomalous words. As the fixation duration and N400 effects are associated with each other [2] and react to the same manipulations, it was expected that the fixation duration effect of the anomalous word neighbour would be similarly attenuated (similar to the N400 effect) when compared to unrelated anomalous words. Further, since P600 is thought to reflect an integration phase in sentence comprehension [3], we expected that unrelated anomalous words would generate stronger P600 responses than anomalous neighbours of a plausible word. We also considered the possibility that the semantic responses would be delayed in the case of the anomalous word neighbours of the plausible words.

### Methodological considerations when studying brain activity during reading

RSVP (rapid serial visual presentation) is most common stimulus presentation procedure for studying brain function during reading. In RSVP, each word in a sentence (or other text material) is usually presented on the screen individually one word at a time in a sequence to the participant’s fovea, and the brain activity is time-locked to each word (screen) onset. Arguably, the elicitation of ERPs by individual words makes RSVP an attractive approach to study reading-related brain processes. Indeed experiments utilising the RSVP technique have formed the foundation for the knowledge about brain function during word recognition and higher-level semantic processing. However, the way in which RSVP oversimplifies reading into a static linear process decreases its ecological validity considerably. From eye-movement studies we know that reading consists of multiple dynamic processes, such as visual intake during fixations, the lengths of which are modulated by the processing demands, skipping of words because of their predictability and visual characteristics and frequent gaze regresses to previous parts of the text to re-read material that are ambiguous or hard to understand [4]. Generally, in RSVP experiments presentation times are static, and there is no chance of skipping words or returning back in the text. However, during normal text reading, the parafoveal information of the words surrounding the fixated words is also processed to some extent [4], which is not possible in the standard implementation of RSVP. Alternative versions of RSVP have been proposed to counteract these issues: self-paced RSVP, where [30] the presentation rate of the words is controlled by the participant, and RSVP with flankers [31], where the sentence ‘slides’ over the participants’ foveal field of vision. However, these modified versions also have some problems. Self-paced RSVP arguably requires conscious monitoring of the presentation rate by the participant while eye movements during reading do not have such requirement, as they are largely automatised. Meanwhile, RSVP with flankers requires suppression of the eye movements towards the flankers, which is a cognitive requirement that is not present during natural reading situations. Furthermore it has been shown that pre-saccadic attention prepares the visual system for the next retinal input [32,33]. As pre-saccadic attention is tightly related to impending saccade execution, its role cannot be fulfilled if saccades are not made. Recent findings illustrate that volition in attention allocation and saccade generation towards words facilitate word recognition in a way that is not present in RSVP [34]

Co-registering EEG and eye tracking represent an ecologically valid alternative for studying reading. This is achieved through analysing the EEG signal based on selected gaze behaviours, for example, fixation on a target word. The combination of co-registration and a behaviour-based analysis approach allows the participant to proceed in the reading task at his or her own pace, with the ability to regress back in the text as well as to skip words. From this participant-initiated reading behaviour we can extract FRPs (fixation-related potentials), electrical brain responses that are very similar to visually evoked potentials from more traditional ERP experiments [34,35]. Indeed, studies comparing the RSVP and FRP methodologies have shown that brain activity during naturalistic FRP experiments differs drastically from RSVP equivalents [1,34], which further emphasises the need for brain research during reading with naturalistic experimental designs.

When performing reading studies with the FRP method, two things need to be considered. First, the ocular artefacts and extra-ocular muscle artefacts feature prominently in the co-registered EEG signal [35]. Generally, these artefacts are stronger than the signal of interest and thus need to be removed from the data. This can be achieved with blind-source separation methods such as ICA and temporal selection methods [36,37]. Temporal selection exploits the dependence between the ocular artefact independent component time course and the time course of the optical eye-movement record [37]. Recent research has shown that artefact signals associated with FRPs can be effectively managed [1,35,38,39]. Second, a more complex issue relates to the fact that during reading fixations occur in relatively fast succession, which results in additional spatiotemporal mixing of the scalp-recorded EEG/ERP signals, especially in the parts of the signal that exceed the duration of the fixation that is used as the time-locking event [40]. As cognitive processes have an evident influence on eye movements [4], changes in gaze pattern due to differences in conditions reflect the mixing of brain activity from different sources in the latter parts of the averaged epoch [40]. We propose to untangle this issue by analysing the time courses of these underlying activity patterns by separating them with blind-source separation methods, such as ICA [36,41].

Even though identifying and removing artefactual signals with ICA from the signal of interest is the typical use in contemporary EEG analysis [42], ICA can also be applied to separate brain signals from each other. This can be of particular interest for late latency components, which typically have multiple neural sources, or when there are multiple overlapping sensory responses. The underlying assumptions of ICA place certain restrictions on the nature of the sources detected. First, a signal arising from a spatial source is assumed to be temporally independent from other spatial sources [36,41]. Second, the spatial source is assumed to be in a fixed location throughout the duration of the measurement of the data of interest [43]. As independent components are defined as spatially fixed sources that change in activity through time, several interesting possibilities arise. First, there is the option to forward project an independent component’s activity to a scalp activity and localise it [43,44]. Second, data from several experiments from the same subject can be inserted into the same decomposition to determine whether different experiments share psychological processes and underlying neural mechanisms [42]. Third, a decomposition analysis (run) can also include data from multiple subjects, and thus the obtained group solutions can be applied to all of the subjects [45]. Hence, the application of ICA beyond artefact cleaning is a promising approach for FRP experiments on natural reading, as the stationarity of sources provides a solution to the ever-present spatio-temporal overlap of activity from previous and consequent fixations and their potential confounds to the scalp signal. Running ICA across a sample of participants also reduces measurement-related error variation (e.g. due to random individual differences in arousal state) and should thus improve the results of ICA decomposition when looking for shared brain activity sources across the sample. In the current study, we employed a group ICA procedure to disentangle spatially overlapping processes during sentence reading and when encountering semantic anomalies in the sentences.

## Methods

### Participants

A total of 66 typically reading elementary school students in grade 6 (from 12.0 to 13.5 years; 36 female) were recruited as part of the eSeek!–Internet and Learning Difficulties: A Multidisciplinary Approach for Understanding Reading in New Media–project. In agreement with the Declaration of Helsinki, the study was approved by the Ethical committee of the University of Jyväskylä. Written consent was acquired from the parents of the participants, and the children had the opportunity to discontinue the experiment at any time. All participants reported normal or corrected vision, no history of learning difficulties or neurological abnormalities and were rated higher than the weakest 13% of the whole eSeek population (whole norming population being 542 children of the same age group) in reading fluency. Reading fluency was estimated with three tests and reduced to a single factor with principal axis factoring with PROMAX rotation using the IBM SPSS 24 statistics programme (IBM Inc.). The three tests were the *Word Identification Test–*a subtest of the standardised Finnish reading test ALLU [46], the *Word Chain Test* [47] and the *Oral Pseudoword Reading Test* [48].

### Apparatus

The experiment was administered using Experiment Builder (1.10.1630) software running on a Dell Precision T5500 workstation. Eye movements were recorded with a table-mounted Eyelink 1000 eye tracker with a 2000 Hz upgrade (SR Research Ltd). Both eyes were recorded at 1000 Hz. The EEG was recorded with a NeurOne amplifier (Bittium) at a 1000 Hz sampling rate with a 128 channel electrode net (Electrical Geodesics Inc.) using Ag/AgCl electrodes. The synchrony between the eye movements and EEG measures was established with a combination of triggering Ethernet messages and transistor to transistor logic (TTL) pulses, both originating from the workstation running the experiment. The stability of the synchronisation between the eye-tracker recording and EEG was checked by comparing the time differences in the trial onset and offset messages in both data streams. The participants leaned on a chinrest while their eye movements were recorded, with a distance of 60 cm from the participants’ eyes, and the EEG was recorded simultaneously. The participants’ responses were recorded into the EEG event stream and into an individual response file on the workstation running the experiment. The experiments were carried out in a dimly lit and soundproofed room at the laboratory facilities of the University of Jyväskylä.

### Stimulus procedure

Before each trial, a black dot appeared on the left side of the screen at the vertical level of the participant’s eyes, and the participant was instructed to remain fixated on it while the experimenter approved the fixation. The black dots also served as confirmation for the validity of the calibration. If the fixation on the dot differed from the calibration by more than 1 degree, the experiment software alerted the experimenter, and the calibration was redone. After the experimenter accepted the fixation, the fixation dot disappeared, and the sentence appeared. The participants were instructed to read the sentence as quickly as possible and then judge whether it was sensible or not by pressing the left button for ‘yes’ and the right for ‘no’ on a two-button response box using the right index and middle fingers. There was no time limit for responding, and the sentence disappeared only after the response. The experiment was divided into four blocks, between which the eye tracker was recalibrated and the quality of the EEG maintained.

### Materials

**Fig 1:**
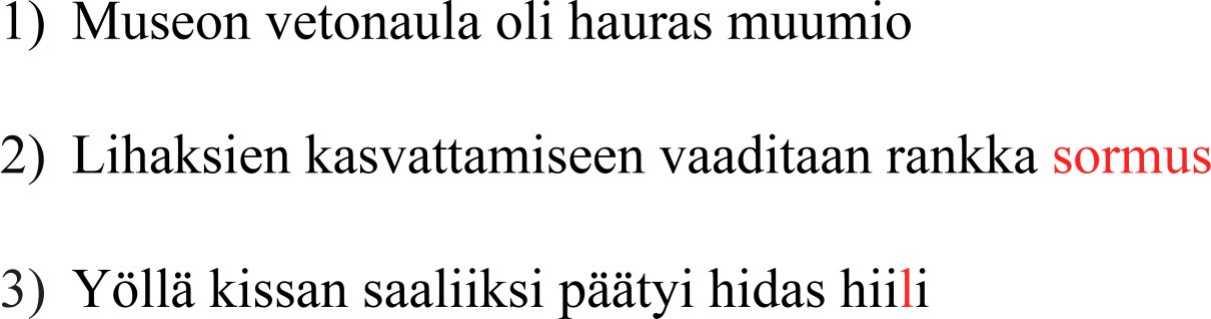
Example stimuli. The part of the sentence that deviates from the context is highlighted in red. Sentence translations: 1) plausible–‘Main attraction of the museum was the fragile mummy’; 2) unrelated anomalous–‘For building up muscle mass one needs an intense ring’; 3) anomalous word neighbour–‘During the night the cat caught a slow coal’ (‘hiili’, coal, being a neighbour of ‘hiiri’, a mouse).

The stimuli consisted of 200 sentences with a median length of six words (ranging from 5 to 9). On the screen, one letter subtended 0.4 degrees visual angle. The sentences were divided into three categories: 100 plausible sentences, 50 sentences where the target word was severely anomalous to preceding sentence context and 50 sentences where the target word was severely anomalous to the preceding context but was an orthographic word neighbour of a plausible word. The categories were balanced in this way to make task response (yes/no) probability 50%. Examples of the sentences are presented in Fig 1. The frequency of the target words and the previous words was controlled for (2x3 repeated measures ANOVA: previous word vs. target word p>0.05, condition p>0.05, condition * previous word vs. target word p>0.05). The frequencies of the target and previous words (Table 1) were extracted from a newspaper corpus [49]. A norming study was completed with an independent sample (N = 10) to estimate the cloze probability of the target words and plausibility of the whole sentences. Also the plausible ending of the sentences in the anomalous word neighbour condition was included in the norming study. Cloze was estimated with a standard cloze task while plausibility was estimated using a seven-point Likert-type scale, where 1 corresponded to ‘not at all plausible’ and 7 to ‘highly plausible’. Unrelated anomalous words did not significantly differ from anomalous word neighbours in either cloze or plausibility (independent samples t-test, p>0.05). The plausible condition did not significantly differ from the plausible ending of anomalous word neighbour sentence in cloze or plausibility (p>0.05). These norms are presented in Table 1.

**Table 1:** Target word and pre-target word frequencies and sentence characteristics.

Condition	Previous Word (SD)	Target Word (SD)
PLA	7.42 (21.58)	7.51 (20.86)
URA	4.98 (6.95)	3.32 (3.59)
AWN	3.62 (4.03)	4.59 (7.27)
	Cloze probability	Plausibility
PLA	41% (36%)	6.76 (0.35)
URA	0 (0)	1.20 (0.23)
AWN	0 (0)	1.26 (0.45)
P-AWN	51% (39%)	6.86 (0.18)

Frequencies (mean incidence in 1 million words) of target and preceding words (with standard deviations). Plausibility: 1 = Not at all plausible, 7 = Highly plausible

Note. PLA = Plausible, URA = Unrelated anomalous, AWN = Anomalous Word Neighbour, P-AWN = Plausible Word Neighbour of the used anomalous Word Neighbour.

### EEG and eye-tracking data preprocessing

Data were preprocessed using Eeglab 13.3.2 [36] with the EYE-EEG extension [35]. The saccades were detected from the gaze location data with a median velocity-based algorithm [50], using 6 standard deviations from the median velocity as a threshold for a saccade (minimum duration of 4ms; if the two saccades were less than 50ms apart from each other, they were merged into a single saccade).

EEG data were off-line filtered, with a high-pass filter of 0.5 Hz and 20 Hz as the low-pass filter. Electric manifestations of eye movements (ocular artefacts) were modelled with ICA, with a PCA (principal component analysis) reduction of the 128 channels to 100 principal components prior to the ICA training. The components were selected to correspond with the optical recording of eye movements using the temporal covariance criterion [37] of 1.1. Selected independent components (ICs) were pruned out of the EEG data.

After the ocular artefact pruning, the EEG epochs with the fixations of interest were selected. These included the first fixation on the target word and the previous fixation. If the previous fixation was not on the word preceding the target word, the whole trial was discarded to make the baseline segments of the FRP similar. Epochs for averaging were -100ms to 900ms, time-locked to the fixation onset. Trials with both correct and incorrect answers were kept. Fixations and their corresponding epochs were discarded if the EEG during the averaging epoch differed by more than 5 standard deviations from the mean on any channel or if the trial ended within the 900ms after first fixation on the target word to avoid confounding activity from trial offset.

**Fig 2:**
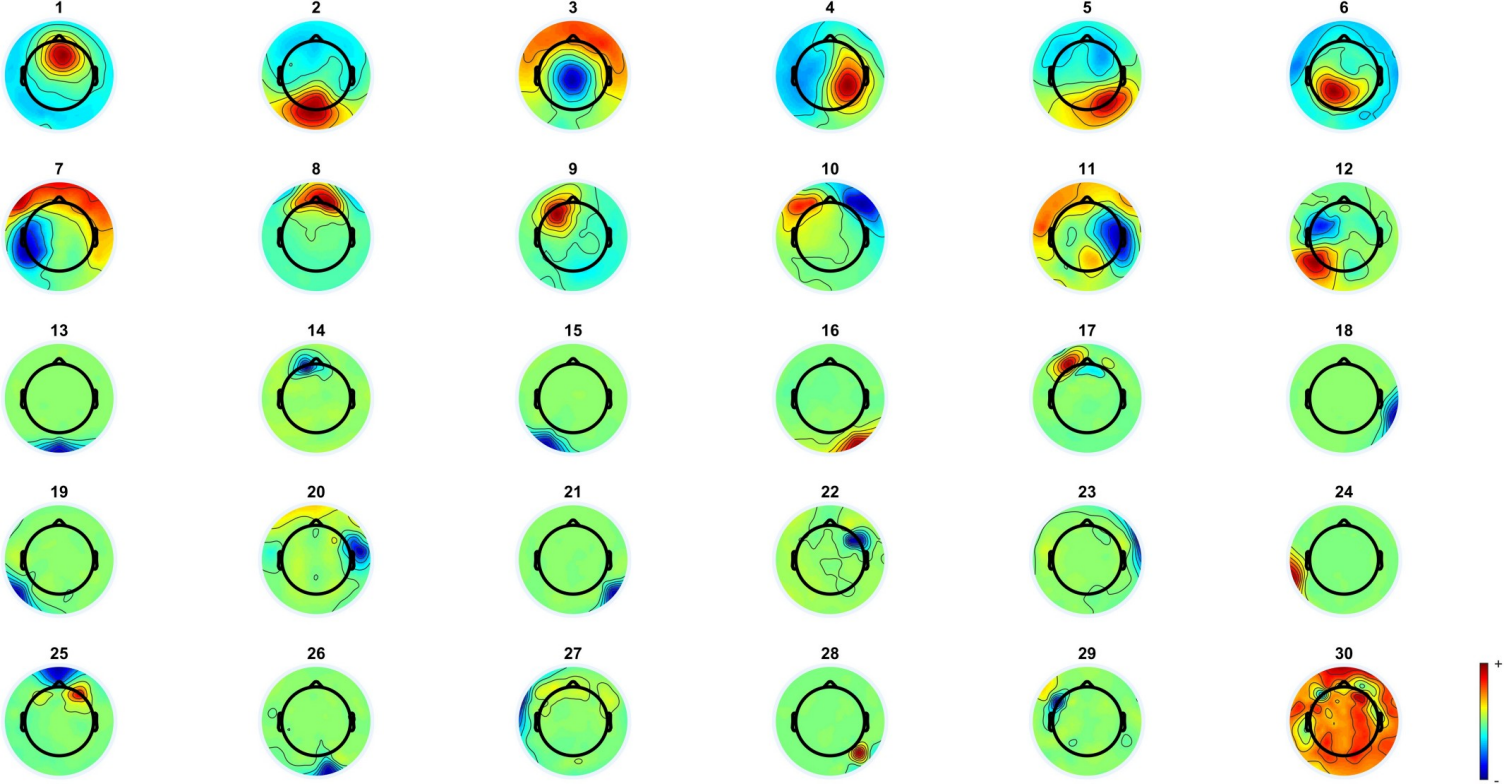
The group-level ICA estimated spatial filters. Components 1, 2, 3, 4, 5, 6, 7, 9, 11 and 12 were retained in the data because they were judged to have a dipolar field structure and thus were likely be generated by brain tissue. Components 8 and 10 were pruned out of the data since they were judged to be likely to have been caused by eye movements. Components 13, 14, 15, 16, 17, 18, 19, 20, 21, 22, 23, 24, 25, 26, 27, 28, 29 and 30 were pruned out of the data because they were judged to be random pop-out artefacts and mechanical artefacts mostly constrained to few channels.

Segmented single trial data (-100ms to 900ms) were subjected to a second run of ICA to determine shared fixation-related components. Prior to ICA, the data for each subject were standardised (basic z-score conversion) so that individuals with strong voltages would not drive the ICA decomposition. The data were downsampled to 250 Hz to make computational requirements feasible, and the group matrix was whitened to 30 principal components with PCA. Extended Infomax, an ICA algorithm sensitive to sub-Gaussian distributions [51], was used because it has been shown to produce good results with non-simulated group EEG data [52]. The resulting group-level ICA weights were then applied to the 1000 Hz individual data, and the resulting IC activities were used for statistical inference. It is important to note here that our application of group-level ICA does not attempt to reconstruct topographies of individual subject’s ICs but applies the group-level weights obtained from the population-mixing matrix directly to the individual subject data. Ten of the 30 components were determined by visual inspection to be such that they could be generated by a cortical source by displaying a dipolar pattern. Pattern was judged to be dipolar if it had either 1-pole uniform field on several adjacent channels or 2-pole field on several adjacent channels and nearby polarity reversal. Components judged to be noise were pruned out of the data if they displayed known artefact characteristics (eye movements, single channel pop-out artefacts). Topographies of all components derived with the group ICA are presented in Fig 2. Table 2 contains the PVAF (percentage of variance accounted for) values of each component calculated with the EEGLAB [36] function *compvar*, PVAF is a measure that describes the amount of variance an independent component explains.

**Table 2:** Descriptive values of the percentage of variance accounted for (PVAF) by the group ICA components.

	Mean PVAF	Sd	Median PVAF		Mean PVAF	Sd	Median pvaf
All IC	82.52%	8.08%	85.44%	Cleaned scalp (*)	51.75%	20.69%	58.4%
IC 1 *	7.97%	5.19%	8.28%	IC 16	1.48%	3.3%	0.63%
IC 2 *	6.93%	4.94%	6.82%	IC 17	1.42%	2.8%	0.74%
IC 3 *	7.24%	5.05%	6.97%	IC 18	1.5%	5.07%	0.54%
IC 4 *	6.76%	4.03%	6.41%	IC 19	1.25%	2.08%	0.61%
IC 5 *	5.74%	3.25%	5.97%	IC 20	1.04%	0.74%	0.88%
IC 6 *	3.98%	2.24%	3.96%	IC 21	1.17%	3.29%	0.37%
IC 7 *	4.21%	5.01%	3.44%	IC 22	1.05%	3.24%	0.46%
IC 8	4.53%	6.63%	2.8%	IC 23	1.02%	1.05%	0.64%
IC 9 *	3.9%	4.08%	3%	IC 24	1.29%	5.36%	0.32%
IC 10	3.35%	3.28%	2.57%	IC 25	1.03%	1.4%	0.63%
IC 11 *	2.57%	1.14%	2.59%	IC 26	1.15%	3.95%	0.25%
IC 12 *	2.46%	6.52%	1.52%	IC 27	0.88%	0.88%	0.56%
IC 13	1.94%	3.76%	0.8%	IC 28	1.56%	10.65%	0.18%
IC 14	1.7%	4.3%	0.9%	IC 29	0.89%	1.92%	0.35%
IC 15	1.76%	4%	0.52%	IC 30	0.76%	1.1%	0.35%

PVAF (percentage of variance accounted for) values for each component.

Note.

* Denotes components retained in the data for statistical analysis

The average quantity of fixations and the corresponding epochs for averaging for each condition were as follows: plausible 83, unrelated anomalous 41 and word neighbour anomalous 41 fixations.

### Statistical analysis of the eye-tracking and response accuracy behavioural variables

**Fig 3:**
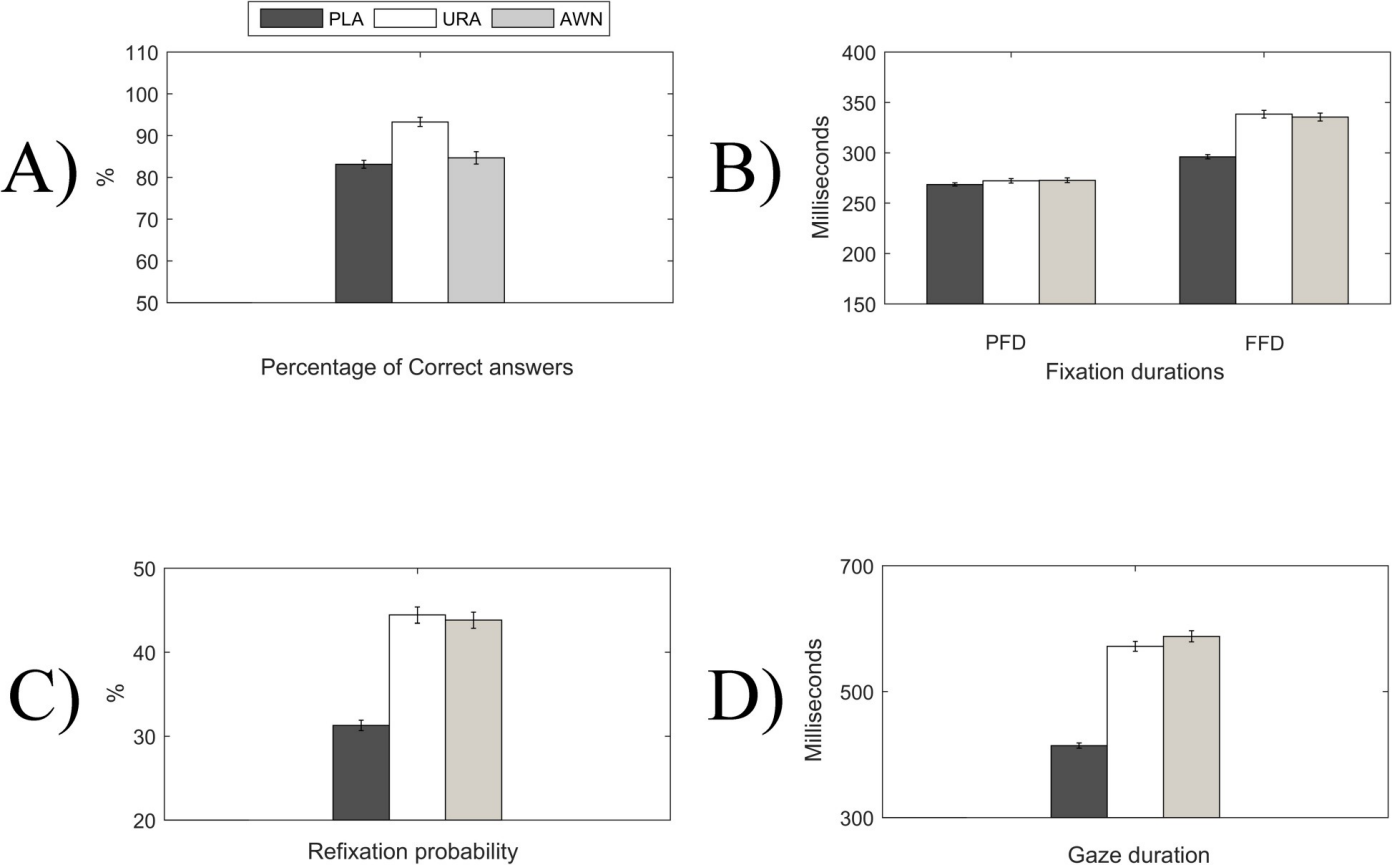
Behavioural measures. Bar graph presentation of the behavioural measures; exact values are presented in Table 3. A) Percentage of correct answers, B) Fixation durations of the previous fixation (PFD) and the first fixation (FFD) and C) Re-fixation probability (RFP) after the first fixation on the target word in a semantic sensibility judgement task in a sentence reading context of 12- to 13.5-year-old children (N = 66). The error bars denote 1 standard error. PLA = Plausible, URA = Unrelated anomalous and AWN = Anomalous word neighbour.

The behavioural variables were analysed in MATLAB. The effects of the conditions on response accuracy were analysed using a Wilcoxon sign-rank test, from which we report Z-values, P-values and effect sizes [53]. Based on the eye-movement variables, the previous fixation duration (PFD), the first fixation duration, gaze duration and immediate re-fixations were analysed. Because trials that were ended with a participant response within 900ms of the first fixation onset were discarded, the re-fixation measure here is a binary measure of regression to earlier parts of the sentence vs. immediate re-fixation within the target word. The eye-movement variables were analysed with the linear mixed effects (LME) models (for FFD and GD) and generalized mixed effects (GLME) models (for refixations), which is the standard procedure in eye-movement research to control for random effects from the individual subjects and items (*fitlme* & *fitglme* functions of the MATLAB statistics and machine learning toolbox). In the LME results, we use the plausible (PLA) condition as a reference condition, against which we compare unrelated anomalous (URA) and anomalous word neighbour (AWN). The estimated random structure was simple intercept structure for trials and subjects. Possible differences between URA and AWN are explored by comparing whether their effect parameters are different (*coefTest* function of the MATLAB statistics and machine learning toolbox). The mean values of the eye-movement variables are presented in Table 3 and also in Fig 3.

**Table 3:** Eye-movement results.

Condition	PFD (SD)	FFD (SD)	GD (SD)	RFP (SD)
PLA	269ms (120ms)	296ms (158ms)	415ms (292ms)	31% (46%)
URA	272ms (115ms)	338ms (201ms)	571ms (410ms)	44% (50%)
AWN	273ms (118ms)	336ms (208ms)	588ms (468ms)	44% (50%)

Means and standard deviations of the previous fixation duration (PFD), the first fixation duration (FFD), gaze duration (GD) and re-fixation probability (RFP).

Note. PLA = Plausible, URA = Unrelated anomalous and AWN = Anomalous word neighbor.

### Statistical analysis of the first fixation time-locked FRPs

FRPs were analysed with nonparametric cluster-based pairwise permutation statistics in Besa statistics 2.0 (for the method description, see[54]), which provides a solution to the multiple comparison problem present in multi-channel EEG recordings. Nonparametric cluster-based permutation tests have two essential steps. First, the desired test (here normal t-test) is run over all channels (when done at the scalp level) and time-points. Then, values over a certain significance (here the typical p<0.05 threshold) are clustered based on clustering criteria. Here, these criteria were temporal adjacency (significant samples were consecutive) and an electrode distance of 3cm (a significant sample in the electrode was part of a cluster if the distance from another significant sample in another electrode was less than 3cm). All t-values of the clusters (each sample, each electrode) are summed to form the cluster test statistic, which is used to estimate test significance. Second, the distribution to estimate this test statistic is generated by randomly re-assigning the condition labels in each average and running the test and clustering procedure again and storing the results in the permutation distribution. When the real observed sum-t probability in contrast to permutation distribution is smaller than 0.05, then the observed cluster is considered to be statistically significant. We used 10,000 permutations to define the permutation distribution. With three conditions, the pairwise condition contrasts for the permutation statistics were as follows: unrelated anomalous vs. plausible, word neighbour anomalous vs. plausible and unrelated anomalous vs. anomalous word neighbour condition. For the sake of simplicity, when we use the terms negative or positive in the results section, they describe the amplitude difference between the first response and the second response in the paired comparison.

## Results

### Response accuracy

The mean response accuracy (percentage of sentences correctly identified as sensible/insensible) was 83.34% (SD = 7.27) for the plausible condition, 93.4% (SD = 8.82) for the unrelated anomalous condition and 84.86% (SD = 12.02) for the anomalous word neighbour condition. Unrelated anomalous sentences had a significantly higher response accuracy (RA) than plausible (Z = 5.525, p<0.000001, r = 0.481) or word neighbour anomalous (Z = 6.414, p<0.000001, r = 0.558) sentences. There was no difference in response accuracy between plausible and anomalous word neighbour sentences (Z = 1.028, p = 0.303, r = 0.09).

### Eye-movement results

#### Fixation durations

There were no significant effects of condition between plausible (PLA), unrelated anomalous (URA) and anomalous word neighbour (AWN) in PFDs (intercept: beta = 269.77, SE = 5.70, t = 47.31, p<0.00001, CI = 258.6–280.95; URA: beta = 3.62, SE = 4.12; t = 0.88, p = 0.37865, CI = -4.44–11.70 AWN: beta = 3.69, SE = 4.11; t = 0.89, p = 0.37042, CI = -4.38–11.76 and URA vs. AWN (p = 0.9892)). Analysis of FFD (intercept: beta = 295.49, SE = 7.29, t = 6.71, p<0.00001, CI = 281.19–309.78) showed that FFD was longer for URA (beta = 44.613, SE = 6.65, t = 6.71,p<0.00001, CI = 31.58–57.64) and AWN (beta = 40.98, SE = 6.65, t = 6.16, p<0.00001, CI = 27.94–54.01) target words than for PLA target words. There was no significant difference between the FFD for the anomalous word neighbour and unrelated anomalous target words (p = 0.6361).

#### First-pass gaze duration

Pattern of results for the first-pass gaze duration was the same as for the first fixation duration (FFD) (Intercept: beta = 411.17, SE = 21.50, t = 19.13, p<0.00001, CI = 369.04–453.30; URA: beta = 164.74, SE = 17.54, t = 9.39, p<0.00001, CI = 130.35–199.13; AWN: beta = 177.45, SE = 17.55, t = 10.11, p<0.00001, CI = 143.05–211.84; and URA vs. AWN (p = 0.5308).

#### Re-fixation probability (RFP)

**RFP** was higher after the first fixation on the unrelated anomalous target word (Intercept: beta = -0.9227, SE = 0.1075, t = -8.58, p<0.00001, CI = -1.1334– -0.7119, URA: beta = 0.6697, SE = 0.0894, t = 7.49, p<0.00001, CI = 0.4945–0.84497), and the RFP was higher after the first fixation on the anomalous word neighbour (AWN: beta = 0.62827, SE = 0.0895, t = 7.02, p<0.00001, CI = 0.4529–0.80365) than after the first fixation on the plausible target word. There was no difference in the RFP after the first fixation between the unrelated anomalous and anomalous word neighbour (p = 0.6858).

#### Semantic anomaly and word neighbour effects in FRPs

**Fig 4:**
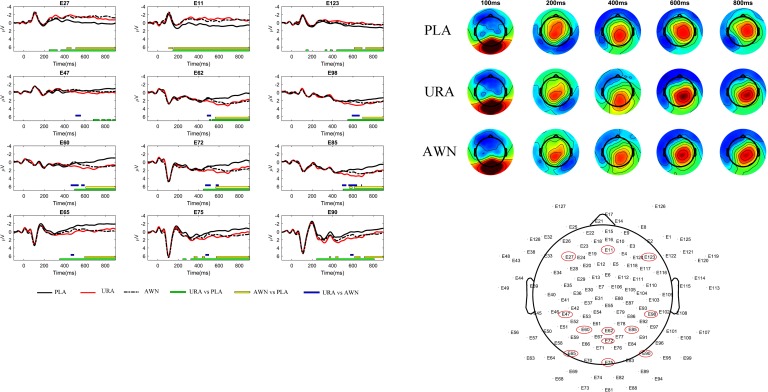
FRPs to the target words. Left Panel: FRPs from the first fixation on the target word on selected channels (E11, E27, E47, E60, E62, E65, E72, E75, E85, E90, E98 and E123 of the GSN-Hydrocel 128-channel cap (Electrical Geodesics Ltd)). Coloured bars under each channel highlight time-points with significant differences that belong to a cluster in the nonparametric permutation test. Right Panel: the associated topographies (all channels) over time in 12- to 13-5-year-old children (N = 66). The zero time-point is the onset of the first fixation on the target word.

**Fig 5:**
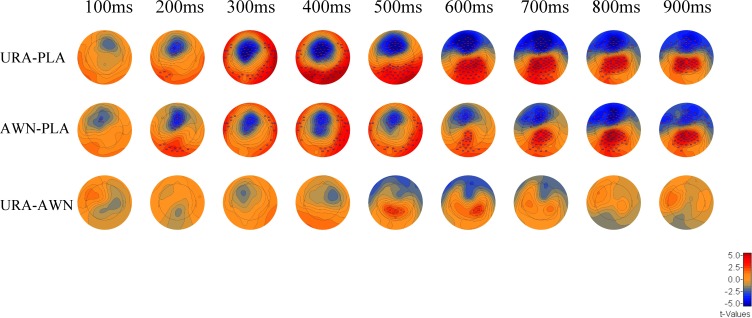
Statistical differences in the FRP amplitude for the first fixation on the target word. The scale depicts differences between the first element of the pair to the second element of the pair in values of the test statistic t. Rectangles signify that the sensor displays a statistically significant difference and belongs to a cluster. The colour of the stars within the squares signifies membership in a specific cluster. PLA = Plausible, URA = Unrelated anomalous and AWN = Anomalous word neighbour.

The first FRPs (Fig 4) showed several statistically significant clusters where the FRPs differed between the conditions (Fig 5). The FRPs differed between unrelated anomalous and plausible conditions, which was evident in a negative cluster in the central frontal scalp area (p < 0.0001) spanning from 138ms to 900ms, which remained stable throughout presence of the cluster, and a positive parietal occipital cluster (p<0.001) with a duration of 180ms to 900ms. The cluster was initially observed in the occipital fringe channels, from which it moved to central parietal locations (roughly 450ms onwards). Anomalous word neighbour and plausible conditions were also significantly different, as there was a fronto-central negative cluster from 97ms to 900ms (p<0.00001) that remained on the frontal sites for the duration of its presence and a positive cluster in the posterior occipital scalp area from 154ms to 900ms (p<0.001 which was initially constrained to occipital fringe channels whence it moved to central parietal sites (roughly 550ms onwards). Furthermore, unrelated anomalous and anomalous word neighbour were significantly different conditions, as we detected one cluster in the time window from 457ms to 698ms, which was positive in the centro-parietal regions (p <0.05).

## Interim discussion

The behavioural results suggest clear and strong effects of semantic anomaly, as the FFDs, GDs and RFPs on the target words all show similar results with increased reading time to anomalous target words. Response accuracy was highest for the unrelated anomalous condition than for the responses for the plausible and anomalous word neighbour conditions. It is important to note, however, that the PFDs in the pre-target word do not show any effects of the semantic manipulation. This effectively shows that the processing shares the same trajectory until the first fixation on the target word. The topographical analysis of the FRPs time-locked to the first fixation onset show a systematic pattern of early frontal negativity (at ca 100–900ms) and late parietal positivity (at ca 500–900ms) for both of the anomalous target word categories. The differences between conditions that we observe are long and widespread. This may, in part, also be due to continuous reading being analogous of the RSVP with fast stimulus rates; fast presentation rates have been shown to diminish N400 and P600 amplitudes in adults [18]. The relatively late parietal positivity can fairly reliably be identified as P600. The P600 difference between anomalous word neighbour and plausible conditions was later than for the unrelated anomalous vs. plausible words. This may suggest that semantic processing of the word neighbour anomalous words of plausible words is delayed in relation to the semantic processing of unrelated anomalous words.

The frontal negativity observed for both anomalous conditions begins quite early and lasts through the remainder of the FRP waveform. One possibility is that this pattern is related to the age difference of our subjects compared to most of the N400 literature reporting adult responses. However, support for the age-related shift of the N400 topography in the previous literature is scarce and relatively old [55]. Thus, the argument for developmental differences is suggested here and would require more systematic developmental study with a traditional RSVP experimentation style. Another possibility is that this pattern is due to the difference between RSVP and FRP measurement techniques, although this is not supported by findings from previous research comparing RSVP and FRP in semantic processing [1]. A reasonable line of thought could also be to relate the current results to the evidence of early contributions of the dorsolateral frontal cortex to visual processing [56]. These contributions have been assumed to be of a top-down nature [57,58], and thus one cannot rule out the possibility that these effects are in fact also modulating the fixation durations or saccade targeting (refixation or regression) either directly or indirectly. The possible contribution of saccadic control is supported by the fact that the difference topography is directly on the top of structures that include frontal eye fields (FEFs) [56]. However this cannot be determined by topographical analysis alone. Thus, this claim would gain more support from source analysis and the interpretation would be constrained by providing information regarding the source time behaviour and location estimates.

Significant temporo-spatial mixing can be observed from the difference topographies. For example, the polarity flip of the frontal negativity (the early positivity in the fringe channels) seems to be mixing with the later parietal positivity. Temporo-spatial mixing makes determining the time of the onset of effects difficult, as is evident for example, for the emergence time of the P600 effect, which does not have any distinct or clearly identifiable onset. The spatial aspects of mixing can be counteracted by analysing the time course of the components from the group ICA procedure.

## Group ICA analysis methods

### Statistical analysis of the source activity

Source waveforms were statistically analysed in MATLAB utilising a method similar to the nonparametric cluster-based permutation approach used for the scalp FRPs above [54]. Condition effects were examined with pairwise comparisons using a Wilcoxon sign-rank test sample by sample. The Wilcoxon test was chosen due to the assumption that considering each time-point of the independent component responses to be normally distributed is unreasonable. The multiple comparisons problem was dealt with as follows. Consequent samples (minimum two) with significant differences were defined to be part of the same difference between the signals, and the duration of this difference is what we base our correction on. These ranges consisting of significant differences are later referred to as consequent samples clusters. After this, we employed a randomisation approach, where we flipped the label of the condition within each given subject randomly, re-ran the Wilcoxon sign-rank test sample by sample and stored the length of each cluster that was observed in the permutation distribution. This randomisation step was then repeated 10000 times. If the randomisation iteration resulted in a solution where there was not a single sample with a significant difference, that iteration was entered as zero into the permutation distribution. After the iterations, the permutation distribution contained the distribution of durations of significant differences when the data were randomised. As the null-observation clusters and the true-observation clusters are based on length and contain no negative values, the correction is one-tailed. Those true-observations that were longer than the 95^th^ percentile of the permutation distribution were accepted for further analysis, and those that were shorter than the 95^th^ percentile of the permutation were rejected. In addition to reporting the p-value of the cluster in relation to the permutation distribution, we also report Wilcoxon statistics and effect sizes for the mean of the cluster [53].

### Source localisation

In order to visualise the sources behind the group level ICs, we forward projected components with significant differences after the correction procedure and then used cortically restricted LORETA implemented in Brain Electrical Source Analysis (BESA, Besa GmbH, Gräfelfing, German) using an age-appropriate template MRI for 12-year-olds [59,60].

## Group ICA results

**Fig 6:**
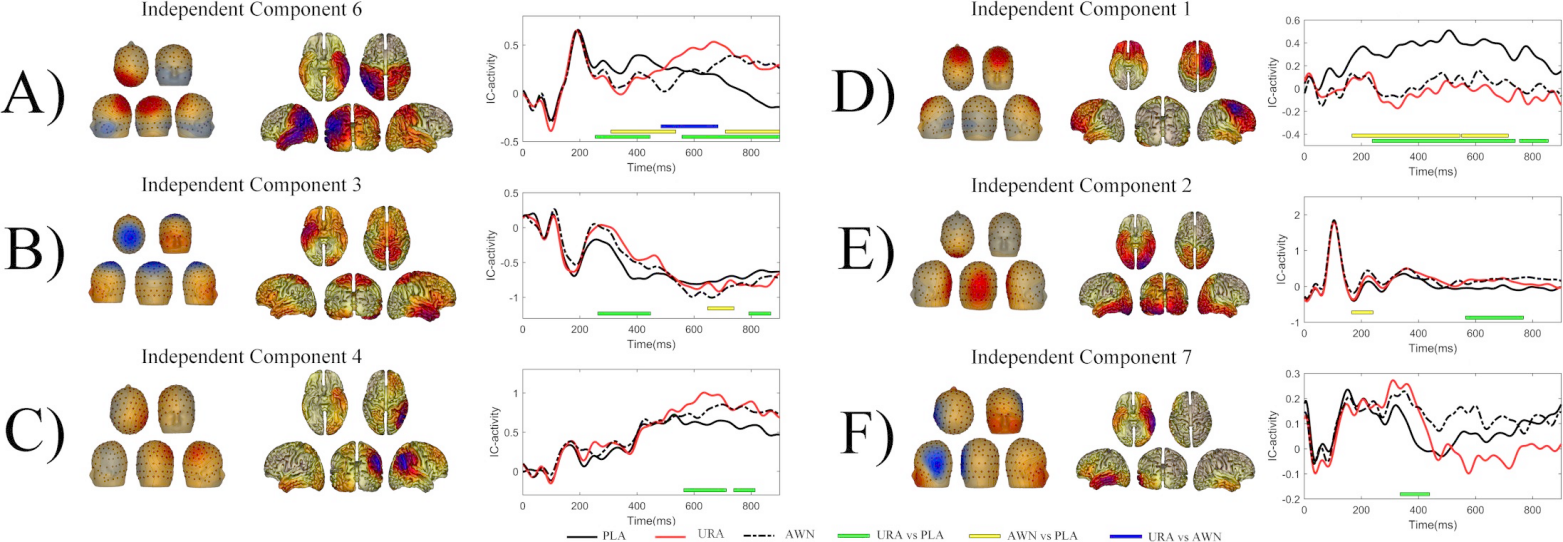
Condition effects in independent components. Topographies of the individual IC spatial filters are shown on the left side of the sub-figure. Red denotes a positive spatial filter weight and blue a negative spatial filter weight. Please note that depending on the activity of the component polarity on the scalp, the sensors change accordingly (for example, in sub-figure A, component 6 is contributing positive voltage to the scalp; however, roughly between 200ms to 500ms anomalous conditions have a more negative–or ‘less positive’–contribution to the measured scalp activity). Cortically restricted LORETA solutions of the individual components are presented in the middle. Temporal evolutions of the components in each condition are shown on the right side of picture (solid black line denoting plausible condition, solid red line unrelated anomalous condition and dashed black line indicating anomalous word neighbour condition); bars under the waveforms denote statistically significant clusters of observations after minimum duration correction (green: unrelated anomalous vs. plausible, yellow: anomalous word neighbour vs. plausible and blue: unrelated anomalous vs. anomalous word neighbour). Acronyms: PLA = Plausible, URA = Unrelated anomalous and AWN = Anomalous word neighbour.

The statistical analysis of source activity indicated significant differences between conditions in components 1, 2, 3, 4, 6, and 7. These components were then subjected to source analyses, the results of which are presented in Table 4 and Fig 6. The statistical results are presented in Fig 6 and in Table 5.

**Table 4:** Source locations of the independent components.

Component	Location
IC 1	Prefrontal cortex, Right
IC 2	Occipital cortex
IC 3	Parietal cortex, Central
IC 4	Parietal cortex, Right
IC 6	Parietal cortex, Left
IC 7	Left temporal cortex

Table 4 summarises the source locations of the independent components that contained significant time course differences after minimum duration correction.

**Table 5:** Statistical results from the independent component comparisons.

Component	Contrast	Cluster onset (ms)	Cluster offset (ms)	Cluster duration (ms)	Cluster duration p	Cluster mean Z	Cluster mean p	Cluster mean R
1	URA-PLA	238	738	501	<0.0001	-4.168	<0.0001	-0.363
	URA-PLA	754	854	101	<0.05	-3.542	<0.001	-0.308
	AWN-PLA	167	547	379	<0.001	-3.600	<0.001	-0.313
	AWN-PLA	551	714	164	<0.01	-2.610	<0.01	-0.227
2	URA-PLA	565	767	203	<0.01	2.661	<0.01	0.232
	AWN-PLA	157	273	117	<0.05	2.986	<0.01	0.260
3	URA-PLA	263	447	185	<0.01	2.891	<0.01	0.252
	URA-PLA	792	869	77	<0.05	-2.495	<0.01	-0.217
	AWN-PLA	648	739	92	<0.05	-2.571	<0.01	-0.224
4	URA-PLA	564	709	149	<0.01	3.338	<0.001	0.291
	URA-PLA	739	813	75	<0.05	2.693	<0.01	0.234
6	URA-PLA	254	445	192	<0.01	-2.776	<0.01	-0.241
	URA-PLA	558	899	342	<0.001	4.124	<0.0001	0.359
	AWN-PLA	309	535	227	<0.01	-3.229	<0.001	-0.281
	AWN-PLA	710	899	190	<0.01	3.804	<0.001	0.331
	URA-AWN	484	683	200	<0.01	3.804	<0.01	0.255
7	URA-PLA	342	434	93	<0.05	2.967	<0.01	0.258

Table 5 summarises statistically significant condition differences in the independent components. Cluster onset is the first time-point where the cluster is present while the cluster offset is the last. Cluster duration is the amount of time-points belonging to the cluster, and the cluster duration p is the p-value category of that cluster duration. Cluster mean Z, P and R are Wilcoxon sign-rank test parameters when the contrast is tested over the average of the cluster time window.

Note. PLA = Plausible, URA = Unrelated anomalous and AWN = Anomalous word neighbour.

## Discussion regarding the semantic anomaly effects in the independent components and their localisation

Group ICA analysis revealed that the differences observed in the scalp had six underlying topographical sources, which differed significantly in their time course in relation to the experimental manipulations. Four of those sources have a plausible contribution to the pattern of early frontal negativity (component 1, Fig 6D) and late posterior positivity (components 4, 3 and 6, Fig 6C, 6B and 6A respectively). Component 7 (Fig 6F), albeit with a brief effect, will be discussed as well as the source location and effect timeframe, which fit well to previous research on semantic processing. One of the components (component 2, Fig 6E) seems to be the manifestation of the early visual response P1 and thus not in the focus of this article. Here, we discuss the frontal source and then the parietal and temporal source observations.

### Frontal projecting component

Frontal negativity (Fig 5), which dominates the difference between conditions in scalp waveforms, seems to be generated by a single IC component (component 1, Fig 6D). Source localisation analyses with cortically restricted LORETA suggest that it is localised in the right posterior frontal middle gyrus, in close proximity to Brodman areas 8 and 6. In PET [61] and some fMRI experiments [62,63], this location has been implicated as an FEF, an area that is associated with many features related to voluntary eye movements, including maintaining fixation, releasing fixation, triggering of eye movements, saccade amplitude and velocity [56] and inhibition of return [64]. In addition, contributions to higher cognitive functions have been proposed, for example, retaining saccade target locations over a couple of seconds, which is essentially a form of working memory [65], and deployment of both overt and covert spatial attention [66]. Transcranial magnetic stimulation of the left or right site of FEF causes saccade latencies to the contralateral direction be altered [56], and cortical electrical stimulation conjugates eye movements to the contralateral direction [67]. Our manipulation is associated with a difference in the next saccade target (refixation vs. regression), which in most cases is in the earlier parts of the sentence, contralateral of the right FEF. Localisation of the early frontal activity to the right FEF, the pattern of saccade targeting differences and previous findings on associated functions of FEF together strongly suggest that the early frontal activity difference that we observe is associated with the first fixation behavioural effects (FFD, refixation probability) that we observed.

### Parietal and temporal projecting components

In the time behaviour of the independent components projecting to the parietal scalp, a biphasic N400/P600-like pattern can be observed. The biphasic N400/P600 pattern appears in two components (components 3 and 6, Fig 6B and 6A, respectively) for both the anomalous conditions. One component (component 4, Fig 6C) only contributes to the P600 effect of the unrelated anomalous condition. As a whole, the parietal brain source structure (in the LORETA analysis) is highly reminiscent of the beamforming results in recent research on semantic and syntactic processing [26], where N400m was localised to left superior temporal and posterior frontal regions and P600m was found to have a plurality of sources, including the bilateral frontal, posterior temporal and parietal regions. In our data, the pattern was similar, with the two components exhibiting a biphasic N400/P600 pattern localised in the left parietal and left posterior temporal areas (component 6) and in the central parietal areas, spreading to the right anterior temporal regions (component 3). Component 4 contributing only to the P600 pattern of the unrelated anomalous condition was localised in the right parietal regions. We will next discuss each of these parts of the parietal pattern in detail.

Component 4 was localised in the right parietal cortex in the approximate area of the right angular gyrus (AG), in the vicinity of Brodman areas 39 and 40 (Fig 5C). AG has been most consistently associated with semantic processing. This is especially the case for the left AG area, although less strong yet equally consistent findings of right AG activation in semantic processing exist as well [68]. Right AG activity has also been found to be associated with cognitive conflict without semantic constraints. This was apparent in research comparing a flanker, stroop and sentence plausibility comprehension tasks, where it was found that the left AG would react to the semantic conflict only while the right AG would respond to all three types of conflict equally [69]. Inhibition of inappropriate responses during no/go tasks has also been associated with the right AG [70]. Thus, in our case, where independent component 4 is contributing only to the P600 pattern of the unrelated anomalous condition, a right AG origin might be related to the capacity of the right AG to process cognitive conflict in several modalities. In the case of the unrelated anomalous condition, the conflict might be gross enough to also be processed in the right AG in the late P600 phase, although the detection of single letter deviation is not strong enough to elicit a significantly different response in the right AG, which is not linguistically specialised.

The independent component 3 topography shows an archetypical centro-parietal N400/P600 complex, and the time course of the component complements this observation (Fig 6B). Unrelated anomalous words significantly modulate both N400 and P600 patterns, whereas anomalous word neighbours modulate only the P600 pattern. The cortical LORETA model shows a pattern of two distinguishable loci of activation: the bilateral superior parietal cortex and right anterior temporal cortex. However, one needs to be particularly cautious when interpreting distributed source model solutions implicating anterior temporal regions, as noise in the frontal and face region scalp sensors tends to migrate to these locations, with a gradual increase of source strength towards the temporal pole being a specific sign of possible artefactual contamination (as stated in discussion with Monto. S. on February 2, 2018, a University researcher at the Centre of Interdisciplinary Brain research at University of Jyväskylä). This is not, however, the case in our data. The locus of the temporal activity is located at the anterior part of the middle temporal gyrus rather than at the anterior pole. If our localisation on this part was actual rather than artefactual, our effects in component 3 could reflect semantic features of the word, as anterior temporal lobes have been argued to act as semantic storage hubs [71]. Anterior temporal lobe contribution to very precise semantic features is particularly evident in semantic dementia, in which bilateral degradation of anterior temporal structures is typical [72,73]. The other areas implicated in our LORETA source localisation solution of component 3 were the bilateral superior parietal areas, which were also implicated in recent research on semantic violations [26].

Component 6 (Fig 6A) contains a highly interesting temporal pattern of activation. First, it displays a contribution to the biphasic N400/P600 for both anomalous conditions. Unlike the other components, component 6 also displays significant differences between the anomalous conditions, and this is essentially because there is delay in the onset of the N400/P600 pattern for the word neighbour anomalous condition. The difference that contributes more negative voltage for the word neighbour anomalous ending than for the plausible ending begins roughly 50ms later than the negative difference in responses between the unrelated anomalous and plausible conditions. Furthermore, the onset of a positive difference between the word neighbour anomalous and plausible conditions occurs roughly 150ms later than the difference between the unrelated anomalous and plausible words. Second, component 6 localises in the LORETA analysis to a broad patch of the left temporal and parietal cortex. Given their heavy involvement in reading and semantic processing in the current experiment, these regions are expected to show activation. For example, the left AG, which is one of the areas implicated in our cortical LORETA model of component 6, is largely involved in all aspects of semantic processing that require concept retrieval and concept integration [68]. Moreover, the left AG has been claimed to provide semantic constraints during language comprehension [74] and to engage when semantic associations are made [75].

The left temporal lobe was also part of the cortical LORETA model of component 6 (Fig 5A). In previous research, the left temporal lobe has been found to be a major contributor to N400 effects in brain lesion, intracranial and MEG studies [24]. These findings converge with fMRI studies, which commonly find middle temporal gyrus activation associated with semantic context manipulations [24]. Overall, the source pattern of component 6 is very consistent with previous research on semantic processing.

In addition, component 7 (Fig 6F) displays a significant difference between URA and PLA around 400ms, and the LORETA model implicates the left temporal lobe as a potential source for activity reflected in the component. However the significance pattern and the waveform of the component make it difficult to interpret how component 7 relates to the pattern observed at component 6. It may well be that the short-lived difference reflects sensitivity to large-scale anomalies but a lack of sensitivity to recognise anomalies of one letter.

The temporal pattern and LORETA solution of the forward-projected independent components converge on an interpretation that the semantic processing of anomalous word neighbours is delayed in relation to the semantic processing of unrelated anomalous words. It could be that the assignment of the semantic role of the anomalous word neighbour becomes delayed because sentence context would favour the semantic role of the plausible word over the anomalous word. The negative contributing cluster is longer for the contrast between the anomalous word neighbour and the plausible word than between the unrelated anomalous word and the plausible word. This could indicate that the assignment of a semantic role or fitting the anomalous word neighbour to the context is more laborious, as the semantic system receives interference from the plausible word that is just one letter away. The delay of onset and longer duration in the earlier negative-contributing phase builds up to a greater delay at the onset of the positive contributing phase. It could be that difficulty in the assignment of the semantic role results in a heavily delayed onset of the integration processes, reflected by the P600 that independent component 6 seems to be contributing to in the later parts of the waveform.

## Summary discussion

Our findings show that when reading sentences with a semantic manipulation of the last word, two types of semantic anomalies (the unrelated anomalous words and the anomalous word neighbours of plausible words) produce similar effects on the FFDs, GDs and the RFP patterns in 12–13.5-year-old children in the grade 6 The FRPs for the unrelated anomalous words share a highly similar morphology with the anomalous word neighbours up to about 500ms after the fixation onset on the target word. The most prominent difference between FRPs for the anomalous and plausible words is the frontal negativity from as early as 100ms to the end of the epoch. The brain source analysis of this negative activation suggested the FEF as a likely source. FEF is an area that has a close relationship with eye-movement execution and contributes to a variety of visuo-spatial attention functions [56]. A parietal positivity, which can be identified as a P600 response, starts to emerge at about 500ms, but only for the contrast between unrelated anomalous and plausible sentence endings. In the responses for the contrast between word neighbour anomalous and plausible sentence endings, this parietal positivity can be observed later, starting at around 700ms. P600 response has been linked to the integration of sentence meaning [3] and a conscious perception of semantic anomalies on the whole sentence level [12]. The time behaviour of the underlying independent components revealed that this delay is also present in an earlier time window around 250ms (see Fig 6A). The source analysis of the components projecting to the parietal scalp areas revealed bilateral contributions from the angular gyri and temporal lobes, both related to semantic processing [24].

The response accuracy was similar to the plausible endings and the anomalous orthographic word neighbours, and it was clearly highest for the unrelated anomalous sentence ending. We propose that the effects of the FFD, GD, RFP and frontal negativity stem from encountering anomalous visual features in relation to sentence context and that the deviation of a single letter from the plausible word is sufficient to elicit these effects. The effects in P600 amplitude and timing, in components projecting to parietal scalp and in response accuracy suggest that the semantic processing of anomalous word neighbour endings is slower than the processing of unrelated semantic anomalies.

FFDs and GDs were longer and re-fixation probability was higher for both the word neighbour anomalous and unrelated anomalous target words than for the plausible target words. Longer GDs for semantic anomalies are typical [6,76], but also FFD effects in relation to semantic manipulations are found in more recent research (see [1,7–10]). The lack of an FFD effect between the unrelated anomalous and word neighbour target words shows that a deviation of a single letter from a plausible word was sufficient to modulate the fixation durations to a similar extent as the anomalous target word without orthographic similarity. In principle, one could expect that processes reflected by the early occipital components P1 and N1 could precede these behavioural effects. However, the absence of P1 and N1 modulations in the current experiment is reasonable since the common modifiers of P1, such as low-level visual features including stimulus size [77], and of N1, such as word frequency [78,79], word length [78] and lexicality [79], were controlled for.

Instead we found early frontal negativity that separates the anomalous conditions from the plausible condition and precedes the gaze behaviour effects. The LORETA source analysis of the frontal independent component resembling the frontal negativity pinpointed the right FEF, an area implicated in eye-movement control [56], as the source of the response, which strongly suggests that the patterns of gaze behaviour and frontal activity are interrelated. FEF also receives fast connections from the primary visual cortex, so it is possible that the effects we observed between conditions are visual rather than semantic and may be related to pre-saccadic parafoveal prediction of post-saccadic foveal input, and thus they may be an effect of visual rather than semantic processing [32,33,80].

In summary, complementary evidence from the FFDs, RFP, GD and frontal negativity suggest that there is a mechanism in the pre-300ms time period that is able to differentiate read words from plausible words on a single-letter level. In all of these variables, the unrelated anomalous and word neighbour anomalous target words differed significantly from the plausible target words.

The effects of semantic context and expectation are systematically found at later latencies, from 200ms onwards from the stimulus onset, and they are reflected in the extensively studied N400 component [11]. Severe semantic anomalies have also been found to elicit P600 responses [3,16]. We can identify P600-like modulation in our scalp waveforms, but the archetypical N400 with central-parietal topography is missing from the scalp waveform. However, analysing the underlying components separately, we can identify two components that project more negative voltages to the parietal scalp between 200–500ms in anomalous conditions and also localise to known N400 generator locations in our LORETA models. Thus, it seems that the frontal negativity that we observe in the scalp waveform is hiding the N400 modulations.

Our N400 results are quite different from those presented in similar RSVP designs in English [14], where N400 was found to be diminished for word neighbours. Authors interpreted that the recognition facilitation from the context spreads to the orthographic information of the neighbouring words. The differences in our results and those of previous RSVP results could stem from various differences in the experimental setups and participants populations–one obvious difference being the utilisation of the FRP technique on our part, which effectively introduces differences in timing between words, parafoveal previews including pre-saccadic attention and volition into the mix. Another plausible reason is the age difference between our participants and those utilised in previous research [14].

In previous research, P600 has been connected to the integrative phase of sentence comprehension [3], response to the impossibility of the sentence [16,81] and syntax anomalies [15]. It has been suggested, however, that rather than signifying the encountering of an anomaly, the P600 effect would reflect re-attending to the complete sentence in an attempt to revise the initial parse of the sentence [3,19–21] or a conscious perception of the semantic anomaly at the whole sentence level [12]. The occurrence of P600 in the anomalous conditions could thus indicate that encountering clearly anomalous words triggers a re-evaluation process for the whole sentence. However, for the word neighbour anomalous condition, P600 seems to have significantly delayed onset, which seems to begin in the earlier N400-contributing phase and accumulate to a more substantial delay in the P600-contributing phase. The accumulated delay of the P600 effect in the neighbour condition could imply that the comprehension process does not orient on the preceding sentence immediately for anomalous word neighbours. The re-evaluation of the preceding sentence could be inhibited by the context, that is, assigning and integrating a semantic role of a plausible word instead of the read anomalous word neighbour target word. The comprehension system might be inclined to consider the deviating letter of the word neighbour as a typographical error and not re-orient the comprehension process immediately to the preceding context. The sentences with neighbour manipulations are mostly considered nonsense at some later point, as indicated by the relatively high response accuracy.

## Conclusion

Based on the data pattern, we arrive at two broad conclusions. First, the observed FFD, RFP, GD and frontal parts of the fixation-related brain response form a pattern that implicates that a divergence of a single letter from a more expected plausible sentence is sufficient to significantly alter gaze behaviour. It is important to note that the difference in frontal activity precedes the difference in gaze behaviour. However, we do not think that these effects are directly related to semantic processing but rather reflect encountering anomalous visual features in relation to sentence context. This is supported by our source localisation of the component resembling the frontal activity in the FEF, a brain area that in addition to making a direct contribution to eye movements also contributes to a variety of visuo-spatial attention functions and can thus be considered a part of the visual processing hierarchy. Further, the very early onset of these frontal effects supports the notion that these effects are related to visual rather than semantic processing.

The second data pattern relates more directly to semantic processing of the anomalous words. The delayed P600 of the scalp waveform for the word neighbour anomalous condition indicates that it takes longer for a single-letter word anomaly to reach the integration stage and perhaps conscious detection compared to a full-word anomaly. The underlying component waveforms indicate that this delay in fact begins earlier in the processing, in the time window corresponding to the archetypical N400. We thus argue that the delay associated with the word neighbour anomalous condition is a result of the difficulty in assigning the semantic role of the word, as it is competing against the semantic role of the plausible word that is just one letter away. This competition is eventually resolved correctly, as can be observed from the delayed but significant onset of P600 for the word neighbour anomalous ending and the relatively high response accuracy for this condition.

### Limitations, boundary conditions and discussion

There are several methodological limitations in the current study. First, to fully control the contributions of parafoveal processing to the process under study, we could have used parafoveal masking of the target word until it was foveated. The hardware that we utilise certainly makes this possible. Forcing parafoveal prediction error with the parafoveal mask would have allowed us to probe whether the pattern that we observed in the frontal scalp and in component 1 was truly a result of a prediction error based on pre-saccadic attention. Then again, it could be argued that including parafoveal masking in our experiment would require us to go through all combinations of mask type with counterbalancing, resulting in nine conditions instead of three, which would have increased the requirements related to the experiment length and population size radically to achieve required signal quality and statistical power. Nevertheless, such a study would be particularly interesting for testing the hypothesis about pre-saccadic attention generating predictions in FEF of the next retinal input during reading.

Second, there is the crucial issue regarding the temporal overlap of activation from previous and subsequent fixations, which effectively creates a situation where one cannot be exactly certain of the degree to which other fixations besides the fixation of interest are contributing to the pattern observed on the scalp. In some studies, this is resolved by a procedure called ‘fixation matching’, where FRPs are matched to eliminate the possible effects of diverging gaze paths. This means that from the condition that has a shorter fixation duration on average, the longer fixations are kept in the FRP, and from the condition that has longer fixations on average, the shorter fixations are selected for the FRP. However, this creates a psychologically biased dataset, as fixation durations and other gaze events are thought to reflect cognitive operations and their processing costs [35]. We thus adopt the position that ‘fixation matching’ compromises the ecological validity of FRP studies, and thus the overlap issue should be addressed with signal processing techniques. We have addressed this issue through a group-level ICA procedure and a separate analysis of the unmixed components. Examination of component 2 (see Fig 6E) highlights the first clear-cut feature that we can observe in the waveforms, the P1/λ-response. This component localises to the primary visual cortex and reflects the initial processing of the low-level features of the foveated stimuli. Thus, if we expect the subsequent fixations to confound the conditions differently, we should observe signs of it in the later parts of the time course of component 2. Of course, this applies mainly to the timing distribution differences between conditions, not to the semantic or other features to which the primary visual cortex is insensitive. From the statistical results of component 2, we see that there is a late difference between the unrelated anomalous and plausible words between 562ms and 774ms. As it is highly unlikely that this difference would reflect semantic processing, we conclude that it is a manifestation of the difference in the fixation timing of the subsequent fixations between conditions. It would take roughly 100ms (from the first positive peak of component 2 to the first positive peak of component 6 and component 4) for effects from this difference in the primary visual cortex to influence the P600 results. All of our results begin prior to this time-point of 660ms. Hence, if the confounding influence is there, it is very minor. If the activity of component 2 would suggest otherwise, the temporal overlap could be further investigated with recently developed signal processing techniques. For example ADJAR (Adjacent Response algorithm)[82,83] or GLM-based linear deconvolution[84] would be suitable (with concrete adjustments to pre-processing pipeline).

Third, we did not rotate sentence frames across conditions, so each target word was always associated with a specific sentence. Therefore, we cannot eliminate the possibility that our results could be affected by random differences between the sentences rather than by our manipulation within these sentences. However, the behavioural results from PFDs argue against this possibility. If the difference between sentences is driving our effects, it should also be present in the PFDs. We do not see any modulation that would signify confounds from the sentences preceding the target words.

Fourth, the target word was sentence final. This is generally not done in eye-movement studies in order to avoid confounds from wrap-up effects that are associated with the final sentence words. However a recent review of ERP studies illustrates that this assumption of a ‘general wrap up’ process at the sentence end is not supported by empirical evidence and suggests that psycholinguistic research has been damaged by the last word target word avoidance dogma [85]. Moreover, given that our focus is on late responses related to semantic processing, where the context provided by the whole sentence is crucial, we think that our placement of the target word is justified. Semantic anomaly manipulation might cause different reactions if placed in earlier parts of the sentence, which would be an interesting research subject.

Fifth, there is confound in the cloze probability of the plausible condition and the plausible word neighbour of the anomalous word neighbour and thus differences between the plausible and two other conditions can partially be due difference in predictability. However the semantic anomaly in the stimuli is clear and thus we consider it to be the driving influence behind the effects. It is important to note that this confound does not impact comparisons between unrelated anomalous and anomalous word neighbour conditions and thus does not impact the main finding of delayed processing of the anomaly in anomalous word neighbour condition.

Finally we studied 12–13.5-year-old children while typical cognitive neuroscience experiments employ university students around 20 years of age. Our sample is a sub-population of the eSeek-project (eSeek! Internet and Learning Difficulties–Multidisciplinary approach for understanding information seeking in new media), and this article essentially describes how school children process semantic information. The pattern of results might change due to developmental effects if this experiment were to be conducted with a representative adult sample.
